# Prevalencia de patología infantil dental y de la mucosa en un servicio de urgencias hospitalario

**DOI:** 10.23938/ASSN.1041

**Published:** 2023-08-28

**Authors:** Montserrat Suárez Ardura, María García-Pola, José Manuel Cuervo Menéndez

**Affiliations:** 1 Área de Gestión Clínica de Urgencias Hospital Vital Álvarez Buylla Mieres Asturias España; 2 Universidad de Oviedo Área de Estomatología Departamento de Cirugía y Especialidades Médico-Quirúrgicas Universidad de Oviedo Asturias Spain; 3 Servicio de Atención Médica Urgente (SAMU) Oviedo Asturias España

**Keywords:** Enfermedades dentales, Mucosa bucal, Infección focal dental, Servicio de Urgencia en Hospital, Niños, Tooth Diseases, Mouth Mucosa, Focal Infection Dental, Emergency Service, Children

## Abstract

**Fundamento:**

La elevada prevalencia de la patología oral infantil incentiva a conocer sus manifestaciones y el carácter urgente de las mismas, objetivo del presente estudio.

**Metodología:**

Estudio transversal que incluyó pacientes de edad <14 años que acudieron a un servicio de urgencias durante un año. Se analizó la relación entre las variables recogidas y la patología oral.

**Resultados:**

Se incluyeron 55 pacientes, 45,5% niñas, edad media 4,11 años (10 días a 13 años). Globalmente, predominó la patología mucosa (74,5%) frente a la dentaria, y el origen infeccioso (54,6%) frente al traumático (14,5%). La patología dentaria (50% odontalgia y 35,7% infecciones) se asoció significativamente a edad >6 años (66,7 vs 10%), dolor (40,7 vs 10,7%) y ausencia de fiebre (37,9 vs 11,5%). La patología mucosa (61% infecciones: 53,7% víricas y 31,7% por herpangina) se asoció significativamente a edad ≤6 años (60 vs 6,7%); y fiebre (76,9 vs 17,2%). Se observaron seis lesiones traumáticas sobre la mucosa y dos en los dientes; significativamente más pacientes acudieron a urgencias antes de 24 horas (mediana =1 hora) que en caso de infección (100 vs 51,7%). La edad ≤6 años se asoció significativamente a fiebre, patología mucosa, infección de la mucosa y herpangina, y la >6 años se asoció a dolor, tratamiento antibiótico previo y al alta y pauta previa de AINE.

**Conclusión:**

La patología oral infantil atendida en el servicio de urgencias analizado fue de la mucosa y origen infeccioso, predominando la dentaria en >6 años y la mucosa en ≤6 años, sin diferencia por sexo.

## Abstract (en)

**Background:**

The high prevalence of oral pathology in children encourages to gain further understanding on their manifestations and urgent nature, objective of the present study

**Methodology:**

Cross-sectional study that included patients aged <14 years of age who attended an emergency department over a one-year period. The relationship between the variables collected and oral pathology was analyzed.

**Results:**

Fifty-five patients were included, 45.5% girls, mean age 4.11 years (10 days to 13 years). Overall, mucosal pathology (74.5%) prevailed over dental ones, and lesions of infectious origin (54.6%) over the traumatic ones (14.5%). Dental pathology (50% toothache and 35.7% infections) was significantly associated with being >6 years (66.7 vs 10%), pain (40.7 vs 10.7%) and absence of fever (37.9 vs 11.5%). Mucosal pathology (61% infections: 53.7% viral and 31.7% due to herpangina) was significantly associated with being ≤6 years (60 vs 6.7%) and having fever (76.9 vs 17.2%). Six traumatic lesions on the mucosa and two on the teeth were observed; significantly more patients attended the emergency room within 24 hours (median =1 hour) than in the case of infection (100 vs 51.7%). Being ≤6 years was significantly associated with fever, mucosal pathology, mucosal infection, and herpangina, and being >6 years was associated with pain, previous antibiotic treatment and at discharge, and previous NSAID regimen.

**Conclusions:**

The analyzed pediatric oral pathologies treated in the emergency department are of mucosal and infectious origin. Dental disease prevail in children >6 years of age and mucosal lesions in those ≤6 years; no sex differences are observed.

## INTRODUCCIÓN

La salud oral está determinada fundamentalmente por la presencia de enfermedades de los dientes y de la mucosa oral en la que intervienen numerosos factores concurrentes a nivel comunitario, familiar e individual^1^.

Los estudios de prevalencia de la salud oral infantil se han enfocado en mayor medida al proceso cariogénico ya que, en esta etapa de la vida, la prevalencia de caries oscila entre el 4,8 y 16,2% en dientes deciduos, y entre el 13,8 y el 27,3% en los definitivos^2^.

Sin embargo, pocos estudios han recogido la prevalencia de la patología de la mucosa oral infantil^3^^,^^4^, con cifras que fluctúan entre el 4 y el 69,5%^4^. Esta gran variabilidad puede estar influida por el tamaño muestral y el área geográfica, así como por otros factores como el ámbito asistencial en el que se realiza el estudio, el diseño metodológico o la patología analizada^5^.

Uno de los cometidos propuestos por la Federación Dental Internacional para el año 2030 es que los profesionales de la salud tengan conocimiento, habilidades y medios para contribuir adecuadamente a la prevención y el manejo efectivos de las enfermedades bucodentales y colaborar en todas las disciplinas de la salud para mejorar la salud y el bienestar^6^. De la literatura se desprende que son escasas las aportaciones en relación al conocimiento de las enfermedades orales en la edad infantil que acuden a un servicio de urgencias, cuya interpretación facilitaría la elaboración de protocolos de diagnóstico y de tratamiento.

En aras de aportar más datos a la propuesta de disminuir el grado de afectación de la patología oral infantil, y teniendo en cuenta la escasez de datos en relación a patologías de la mucosa oral, el objetivo planteado en el presente estudio es determinar la prevalencia y tipo de patología oral entre la población infantil que acude a un servicio de urgencias hospitalario, y los factores asociados que motivan la consulta.

## MATERIAL Y MÉTODOS

Estudio transversal, retrospectivo, realizado en un hospital de segundo nivel asistencial (Hospital Vital Álvarez Buylla, HVAB) localizado en Mieres (Asturias, España) entre el 1 de enero y 31 de diciembre de 2017, siguiendo las directrices STROBE^7^. El estudio contó con la aprobación del Comité de Ética de la Investigación del Principado de Asturias (nº 15/19), preservando la protección de datos según la ley vigente y la Declaración de Helsinki.

Se incluyeron de forma consecutiva los pacientes que acudieron al Área de Gestión Clínica de Urgencias del hospital durante el periodo de estudio y que cumplían los siguientes criterios de inclusión: edad <14 años, asistencia al servicio de urgencia debido a patología de la mucosa oral o dentaria, y con diagnóstico de su enfermedad en el momento del alta hospitalaria codificada en el programa informático SELENE. Se excluyeron los pacientes los que en la codificación de su diagnóstico no consta, o figura “Fuga”, “Marcha sin ser visto”, o “Alta Voluntaria”.

Los pacientes fueron examinados por personal facultativo del servicio de Urgencias. En determinados casos, se solicitó interconsulta al servicio de Pediatría del hospital, cuyo personal examinó a los pacientes para valorar las patologías no traumáticas. La exploración se realizó en la camilla del *box* de pediatría y, ocasionalmente en pacientes más mayores, en el sillón del *box* de otorrinolaringología, bajo luz artificial, utilizando depresor y gasas, y linterna o fotóforo según necesidad.

Los datos de los pacientes se recogieron del archivo informático del programa SELENE y fueron trasladados mediante un cuestionario *ad hoc* a una hoja de cálculo Excel. Se registraron las siguientes variables:

- *demográficas*: sexo (hombre, mujer), y edad (años, ≤6 años, >6 años). La variable edad se dicotomizó porque 6 años es la edad considerada para evaluar los objetivos de la salud oral infantill^8^.
- *hábitos tóxicos*: presencia (sí, no) de consumo de tabaco y alcohol, hábitos considerados de inicio a partir de los 12 años^9^,
- *antecedentes personales patológicos:* alergias, inmunodeficiencias, padecimiento de enfermedades sistémicas (cardio-respiratorias, u otras), y consumo de fármacos crónicos (sí, no);
- *enfermedades orales*: de mucosas o dentarias, infecciosas o traumáticas, u otras (si, no);
- *clínicas*: el tiempo medio de evolución de los síntomas desde el inicio de la dolencia hasta acudir a urgencias y en su forma dicotómica según el valor de la mediana (≤24 horas, >24 horas), consultas previas, derivación del paciente a urgencias por un profesional (sí, no), signo febril (sí, no), sintomatología de dolor (sí, no), pruebas complementarias realizadas (hematológicas, de cultivo, de imagen);
- *farmacológicas*: tratamientos pautados previamente a la consulta de urgencias y al alta de urgencias (antibiótico, antiinflamatorios no esteroideos/AINE, analgésicos);
- también se indicó la necesidad de hospitalización, y la derivación hacia otros centros hospitalarios.

Las variables cuantitativas se describieron con la media, la mediana y la desviación estándar (DE), y posteriormente fueron dicotomizadas. Para el estudio analítico se consideraron las variables de forma dicotómica (presencia/ausencia, o sí/no), acompañadas de la frecuencia y porcentaje. Las relaciones entre variables cualitativas independientes se valoraron con el test Chi-cuadrado de Pearson (χ^2^), o el test exacto de Fisher cuando las frecuencias esperadas eran pequeñas. Las relaciones entre la prescripción de fármacos previa y en urgencias se analizaron con las pruebas de simetría exacta (McNemar) y relativa, aplicando la corrección de continuidad de Yates en el caso de muestras pequeñas. El análisis estadístico se efectuó mediante el programa R (*R Development Core Team*, versión 4.3.1). El nivel de significación considerado fue p ˂0,05.

## RESULTADOS

Se incluyeron 55 pacientes en el estudio; 25 eran niñas (45,5%) y la media de edad fue 4,11 años (DE: 3,61; rango: 10 días a 13 años).

No se registraron hábitos tóxicos ni alergias conocidas. Entre los antecedentes destacaron las enfermedades cardio-respiratorias (14,5%), seguidas por asma (7,3%), y enfermedades dermatológicas (5,5%). Cinco pacientes (9,1%) presentaron inmunodeficiencias: síndrome polimarfomativo (n=2), hipoxias isquémicas (n=2) y una de origen nefrourológico. Cinco pacientes recibían tratamientos crónicos: diuréticos (n=2) e inhaladores (n=3) (Tabla 1).

El tiempo medio de evolución de los síntomas antes de acudir a urgencias fue de 49,52 horas (mediana: 24 horas); cuatro fueron derivados por un profesional (7,3%) y 18 (32,7%) ya habían recibido consulta previamente.

**Tabla 1:** Características de la muestra estudiada

Variables	n (%)
*Demográficas y antecedentes*	
Edad (≤ 6 años)	40 (72,7)
Sexo (varón)	30 (54,5)
Antecedentes personales	20 (36,4)
Enfermedades cardio-respiratorias	(14,5)
Otras enfermedades	10 (18,2)
*Alergias*	
Inmunodeficiencias	(9,1)
Tratamientos previos crónicos	(9,1)
*Clínicas*	
Tiempo de evolución (≤24 horas)	31 (56,4)
Consulta en los días previos	18 (32,7)
Derivado por un profesional sanitario	(7,3)
Fiebre	26 (47,3)
Dolor	27 (49,1)
Pruebas complementarias	(12,7)
*Evolución*	
Ingreso hospitalario	(5,5)
Derivación	(5,5)

Las infecciones fueron la causa más frecuente de consulta (54,6%). La patología de la mucosa motivó el triple de consultas a urgencias que la patología dentaria (74,5 frente a 25,5%; p<0,001).

La principal patología dentaria fueron las odontalgias o dolor dentario (50%); las infecciones supusieron un 35,7%. Por el contrario, los procesos infecciosos motivaron el 61% de las consultas por patología de la mucosa, seguido por la estomatitis aftosa (17,1%) y los edemas (7,3%) (Tabla 2).

La mayoría de infecciones de la mucosa fueron de etiología vírica (n=22; 88%), siendo la herpangina diagnosticada en más de la mitad de esos casos (n=13; 59,1%) (Tabla 2). La infección de la mucosa fue significativamente más frecuente en pacientes ≤6 años (60 vs 6,7%; p=0,012) y con fiebre (76,9 vs 17,2%; p<0,001). La herpangina también fue más frecuente entre las niñas (32 vs 16,7%; p=0,183) y en pacientes que recibieron tratamiento AINE al alta (32,3 vs 12,5%; p=0,087), y menos frecuente entre los pacientes con dolor (18,5 vs 28,6%; p=0,380), aunque de forma estadísticamente no significativa.

En los dientes, se observaron signos de infección clínica en cinco casos (35,7%). La patología dentaria fue más frecuente en pacientes con edad >6 años (66,7 vs 10%; p<0,001), cursando usualmente con dolor (40,7 vs 10,7%; p=0,011) y sin fiebre (37,9 vs 11,5%; p= 0,025).

**Tabla 2:** Frecuencia y tiempo de evolución de patología oral por tipo

Patología	Global n=55	Mucosa n=41 (74,5%)	Dentaria n=14 (25,5%)
*Infecciosa*	*30 (54,6)*	*25 (61,0)*	*5 (35,7)*
*vírica*	22 (40,0)	22 (53,7)	0
herpangina	13 (23,6)	13 (31,7)	0
herpes	6 (10,9)	6 (14,7)	0
enfermedad mano pie-boca	3 (5,4)	3 (7,3)	0
micótica	3 (5,4)	3 (7,3)	0
candidiasis pseudomembranosa	3 (5,4)	3 (7,3)	0
*No infecciosa*	*7 (30,9)*	*10 (24,4)*	*7 (50,0)*
estomatitis aftosa	7 (12,7)	7 (17,1)	0
otras	10 (18,8)	3 (7,3)	7 (50,0)
*Traumática*	*8 (14,5)*	*6 (14,6)*	*2 (14,3)*
Evolución ≤ 24 horas*	31 (60,8)	26 (63,4)	5 (35,7)

*: media (desviación típica), calculado en 51 pacientes.

Se observaron lesiones traumáticas en ocho casos, 6 sobre la mucosa y 2 en los dientes (14,5%). Ningún paciente con lesión traumática acudió con fiebre ni había realizado consultas previas. La media de tiempo transcurrido desde el accidente fue de 4,2 horas (mediana =1 hora), siendo la frecuencia de pacientes que acudieron a urgencias antes de 24 horas significativamente mayor que en el caso de procesos infecciosos (100 vs 51,7%; p=0,021).

En relación a las pruebas complementarias requeridas, a cuatro pacientes con infección (dos de mucosa y dos dentarias), se les solicitaron hemograma y estudio bioquímico, además de pruebas de coagulación a uno de ellos. A otro paciente se le practicó un test rápido de detección de antígenos de estreptococos del grupo A y una ecografía. El hemocultivo se solicitó a otros dos pacientes, uno de ellos con diagnóstico de gingivoestomatitis herpética y otro con infección dental.

Tres pacientes quedaron ingresados en el HVAB y otros tres pacientes fueron derivados al centro hospitalario al que pertenecían.

La prescripción de AINE y de analgésicos fue superior al alta que a la consulta, debido a que las nuevas prescripciones superaron a las suspensiones del tratamiento previo (Tabla 3, prueba de McNemar).

**Tabla 3:** Tratamiento farmacológico previo y al alta en los 55 pacientes del estudio, por tipo de fármaco

	Prescripción		Prescripción		p (McNemar)	p*
Fármaco	Previa n (%)	Alta n (%)	Retirada	Nueva		
Antibiótico	(14,5)	(14,5)	4 (8,5)	(8,5)	1	0,011
AINE	12 (21,8)	31 (56,4)	2 (3,6)	21 (38,2)	<0,001	<0,001
Analgésico	(5,5)	14 (25,5)	0 (0)	11 (20)	0,018	0,882

AINE: antiinflamatorio no esteroideo; *: prueba de simetría relativa, aplicando χ^2^ con la corrección de continuidad de Yates.

El cambio en el grupo de pacientes con prescripción previa respecto al cambio en el grupo de pacientes sin prescripción previa fue mayor para los antibióticos (4/8= 50% vs 4/47= 8,5%), menor para los AINE (2/12= 16,7% vs 21/43= 48,8%) y no significativamente distinto para los analgésicos (0/3= 0% vs 11/52= 21,2%) (Tabla 3, prueba de simetría relativa).

La presencia de dolor se asoció a la prescripción previa tanto de antibióticos (87,5 vs 42,6%; p=0,025) como de AINE (91,7 vs 37,2%; p=0,001). La prescripción al alta de antibióticos se asoció a la presencia de dolor (87,5 vs 42,5%; p=0,025), y la de AINE a la presencia de dolor (71 vs 20,8%; p<0,001) y de fiebre (61,3 vs 29,2%; p=0,018).

Aunque la patología infecciosa fue ligeramente superior en los niños (46,7% vs 44%), la herpangina lo fue en las niñas, como ya se ha comentado. También fue más frecuente en los niños la presencia de fiebre (61,5 vs 48,3%) y la pauta terapéutica al alta de antibióticos (75 vs 51,1%), pero en ningún caso de forma estadísticamente significativa.

Los pacientes ≤6 años presentaron con mayor frecuencia fiebre (88,5 vs 58,6%; p=0,005), afectación de la mucosa (87,8 vs 28,6%; p<0,001), infección de la mucosa (96 vs 53,3%; p<0,001), y herpangina (previamente reseñada). Por el contrario, en los pacientes >6 años fue más frecuente la presencia de dolor (44,4 vs 10,7%; p=0,005), el tratamiento con antibiótico, tanto previo (62,5 vs 21,3%; p=0,016) como al alta (75 vs 19,1%; p=0,001), y la pauta previa de AINE (66,7 vs 16,3%; p=0,001), además de mayor patología dentaria.

## DISCUSIÓN

En el presente estudio se analizó la patología oral como motivo de consulta de una muestra de 55 pacientes con edades inferiores a 14 años que acudieron a un servicio de urgencias hospitalario. En nuestro conocimiento, este es el primer estudio que aporta las características de la patología odontoestomatólogica en la población infantil en un servicio de urgencias de un hospital de segundo nivel asistencial. Previamente, Rancaño-Garcia y col habían descrito que, en una población de edad media de 39,1 años que acudía a los servicios de urgencia de Atención Primaria en el municipio de Oviedo, el motivo de consulta del 3,3% de los pacientes era odontoestomatológico, sin especificar el tipo de patología^10^.

No se ha observado relación entre el sexo y las patologías de forma estadísticamente significativa, ratificando lo descrito en otros ámbitos asistenciales^5^^,^^11^^,^^12^. La media de edad en este estudio es inferior a la de otros estudios realizados en una consulta odontológica universitaria o dermatológica (4,1 vs 6,5 años^5^ y 7,8 años^11^).

Se estima que la estomatitis aguda en los niños es una de las causas más frecuentes que motiva acudir a los servicios de urgencias pediátricos, ocupando el 3,3 por 1.000 de los mismas^13^. Otros estudios mencionan la patología herpética como la patología infecciosa más prevalente en las consultas odontológicas^3^^,^^4^ y en urgencias^13^, seguida de la herpangina y la enfermedad mano-pie-boca^3^^,^^4^, mientras que en nuestra muestra la frecuencia de la herpangina duplicó la de la patología herpética y cuadruplicó la de la enfermedad mano-pie-boca. El diagnóstico de estas tres enfermedades suele ser eminentemente clínico y, por tanto, no precisa de pruebas complementarias, como así ha ocurrido con la muestra presentada. La primoinfección herpética se inicia con un proceso febril y, tras 1-2 días, aparecen vesículas por toda la mucosa, con una distribución generalizada, mientras que en la herpangina se localizan a nivel de paladar blando, úvula y pilares; si estas vesículas intraorales se acompañan de otras en manos y pies, se considera diagnóstico de presunción la enfermedad mano-pie-boca^14^.

La estomatitis aftosa fue la segunda patología más frecuente en nuestro estudio, aunque con una frecuencia inferior a la descrita en otros estudios^15^ (12,7 vs 18,9%) que la señalan como la más común. En ocasiones, los datos de estas estomatitis se enmarcan en el contexto de su recidiva^8^, es decir, de estomatitis aftosa recurrente; sin embargo, debido a la falta de seguimiento de los pacientes, tan solo hemos podido considerar que esta manifestación definida por la erosión no era de origen traumático.

En el presente estudio se ha constatado que el dolor dental es más prevalente en edades superiores a los 6 años al compararlos con la edad inferior (44,4 vs 10,7%), datos que concuerdan con el patrón de la manifestación del dolor recogido en una reciente revisión sistemática, más prevalente en edades entre los 6 y 9 años que en edad preescolar (52 vs 27,7%)^16^.

Las lesiones traumáticas propiamente dichas son frecuentemente observadas en las clínicas dentales privadas, en las instituciones universitarias e incluso en las consultas de Atención Primaria, alcanzando el 22,15%^17^, cifras superiores a las referidas en nuestra muestra (14,5%).

Se ha de destacar el reducido número de pacientes que requirieron pruebas complementarias para su diagnóstico (12,7%). Este hallazgo debería de tenerse en consideración para aquellas situaciones en las que se pretende establecer áreas de visita rápida para reforzar el flujo de los pacientes que acuden a urgencias con menos signos y síntomas de gravedad y que por sus manifestaciones orientan hacia el diagnóstico clínico^18^.

La principal limitación del estudio es la falta de potencia estadística derivada del pequeño tamaño muestral, que ha podido influir en el bajo número de variables relacionadas con las distintas patologías orales. Otra de las limitaciones es la ausencia de aportaciones de radiodiagnóstico de la patología dentaria debido a la carencia en el servicio de urgencias de equipamiento como ortopantomógrafo o de rayos X intraoral, pero que refleja la realidad de nuestro hospital, una realidad frecuente en hospitales de segundo nivel asistencial.

Sin embargo el estudio representa un pilar en la clasificación de los tipos de enfermedades, diferenciando topografía de mucosa y dentaria, así como el probable origen infeccioso o traumático, que pudieran ser el punto de partida de futuras investigaciones para aportar datos más precisos sobre la patología oral infantil. Bajo esta percepción, se aportarían datos para elaborar algoritmos de diagnóstico.

En conclusión, el perfil de la patología oral infantil atendida en el servicio de urgencias del hospital de segundo nivel asistencial analizado, muestra una tendencia hacia la afectación en la mucosa con marcado origen infeccioso en edades ≤6 años, frente a la dentaria, en edades >6 años, sin que hubiese una predilección significativa por uno de los sexos.
